# Cooperative Recognition of Internationally Disseminated Ceftriaxone-Resistant *Neisseria* *gonorrhoeae* Strain

**DOI:** 10.3201/eid2404.171873

**Published:** 2018-04

**Authors:** Monica M. Lahra, Irene Martin, Walter Demczuk, Amy V. Jennison, Ken-Ichi Lee, Shu-Ichi Nakayama, Brigitte Lefebvre, Jean Longtin, Alison Ward, Michael R. Mulvey, Teodora Wi, Makoto Ohnishi, David Whiley

**Affiliations:** World Health Organization, Sydney, New South Wales, Australia (M.M. Lahra); University of New South Wales School of Medical Sciences, Sydney (M.M. Lahra);; Public Health Agency of Canada, Winnipeg, Manitoba, Canada (I. Martin, W. Demczuk, M.R. Mulvey);; Health Support Queensland, Brisbane, Queensland, Australia (A.V. Jennison); National Institute of Infectious Diseases, Tokyo, Japan (K.-I. Lee, S.-I. Nakayama, M. Ohnishi);; Institut National de Sante Publique Québec, Ste-Anne-de-Bellevue, Quebec, Canada (B. Lefebvre, J. Longtin);; Royal Adelaide Hospital, Adelaide, South Australia, Australia (A. Ward); World Health Organization, Geneva, Switzerland (T. Wi);; National Institute of Infectious Diseases, Tokyo (M. Ohnishi);; The University of Queensland Faculty of Medicine, Brisbane (D. Whiley)

**Keywords:** *Neisseria gonorrhoeae*, gonorrhea, ceftriaxone, cefixime, β-lactam, azithromycin, cephalosporin, *penA* gene, penicillin, tetracycline, gentamycin, antimicrobial resistance, ciprofloxacin, spectinomycin, penicillinase, Canada, Japan, France, Spain, Australia, China

## Abstract

Ceftriaxone remains a first-line treatment for patients infected by *Neisseria gonorrhoeae* in most settings. We investigated the possible spread of a ceftriaxone-resistant FC428 *N. gonorrhoeae* clone in Japan after recent isolation of similar strains in Denmark (GK124) and Canada (47707). We report 2 instances of the FC428 clone in Australia in heterosexual men traveling from Asia. Our bioinformatic analyses included core single-nucleotide variation phylogeny and in silico molecular typing; phylogenetic analysis showed close genetic relatedness among all 5 isolates. Results showed multilocus sequence type 1903; *N. gonorrhoeae* sequence typing for antimicrobial resistance (NG-STAR) 233; and harboring of mosaic *penA* allele encoding alterations A311V and T483S (*penA*-60.001), associated with ceftriaxone resistance. Our results provide further evidence of international transmission of ceftriaxone-resistant *N. gonorrhoeae*. We recommend increasing awareness of international spread of this drug-resistant strain, strengthening surveillance to include identifying treatment failures and contacts, and strengthening international sharing of data.

Ceftriaxone is among the last remaining recommended therapies for treating *Neisseria gonorrhoeae* infections and is used in many countries around the world as part of a dual therapy with azithromycin. Cephalosporin resistance in *N. gonorrhoeae* has been associated with modifications of the *penA* gene, which encodes penicillin-binding protein 2 (PBP2), a target for β-lactam antimicrobial drugs (*1*). During 2009–2015, several ceftriaxone-resistant (MIC 0.5–4 mg/L) *N. gonorrhoeae* strains were reported: in 2009, H041 in Japan (*2*); in 2010, F89 in France (*3*); in 2011, F89 in Spain (*4*); in 2013, A8806 in Australia (*5*); in 2014, GU140106 in Japan (*6*); and in 2015, FC428 and FC460 in Japan (*7*). However, until 2017, all of these strains were considered to have occurred sporadically because, except for limited transmission of F89 among persons in France and Spain during 2010–2011, there had been no reports of sustained transmission of these strains identified nationally or internationally. In 2017, this changed, substantiated by independent reports from Canada (*8*) and Denmark (*9*) of gonococcal isolates that had substantive similarity to the previously described FC428 strain in Japan.

The first reported case of the FC428 ceftriaxone-resistant *N*. *gonorrhoeae* strain was in Japan during January 2015 in a heterosexual man in his twenties who had urethritis (*7*). The FC428 isolate was resistant to ceftriaxone (MIC 0.5 mg/L), cefixime (MIC 1 mg/L), and ciprofloxacin (MIC >32 mg/L); susceptible to spectinomycin (MIC 8 mg/L) and azithromycin (MIC 0.25 mg/L); and, unlike all previously described ceftriaxone-resistant strains, a penicillinase-producing *N. gonorrhoeae* (PPNG; MIC ≥32 mg/L) bacterium. The patient was treated successfully with a single dose of spectinomycin 2 g intramuscularly (IM); however, a second isolate with an identical susceptibility profile (FC460) was subsequently cultured from the same patient 3 months later, suggesting reinfection by a separate contact. 

In Canada, during January 2017, a gonococcal isolate (47707) (*8*) of similar susceptibility to the first reported case (including ceftriaxone-resistant MIC 1 mg/L and PPNG; Table 1 [*10*]) was isolated from a sample collected from a 23-year-old woman. This patient had no history of travel, but her male partner, who had been treated empirically and had no culture results available, reported sexual contact during travel in China and Thailand during the fall of 2016. She was successfully treated with combination therapy of a single dose each of cefixime (800 mg orally) and azithromycin (1 g orally) and an additional dose 13 days later of azithromycin (2 g orally). The strain from Denmark (GK124) was also isolated in January 2017, had a similar susceptibility profile to FC428, and was obtained from a heterosexual man in his twenties who had reported unprotected sexual contact with women from Denmark, China, and Australia (*9*). The patient was successfully treated with single doses of ceftriaxone (0.5 g IM) and azithromycin (2 g orally). Here, we report additional FC-428-like cases among persons in Australia, providing further evidence of the sustained international transmission of a ceftriaxone-resistant *N. gonorrhoeae* strain.

**Table 1:** Phenotypic and molecular characterization of ceftriaxone-resistant *Neisseria* gonorrhoeae*

Isolate ID	Year	Country (ref)	MIC, mg/L									MLST	*porB*	*tbpB*	NG-MAST	*penA*	NG-STAR
			CEF	CFM	SPX	TET	CIP	AZM	GEN	PCN	β-lac, PPNG						
FC428	2015	Japan (*7*)	0.5	1	8	0.5	>32	0.25	8	≥32	+	1903	1053	21	3435	60.001	233
FC460	2015	Japan (*7*)	0.5	1	8	0.5	>32	0.25	8	≥32	+	1903	1053	21	3435	60.001	233
GK124	2017	DEN (*9*)	0.5	1	8	NA	>32	0.5	NA	>256	NA	1903	1053	33	1614	NA	NA
47707	2017	Canada (*8*)	1	2	16	4	32	0.5	8	≥256	+	1903	1053	33	1614	60.001	233
A7846	2017	AUS (This study)	0.5	NA	8	2	>32	0.25	4	≥32	+	1903	1053	33	1614	60.001	233
A7536	2017	AUS (This study)	0.5	NA	8	4	>32	0.25	4	≥32	+	1903	9300	21	15925	60.001	233
F89	2010	France (*3**,**10*)	1	2	16	4	>32	1	8	1	–	1901	908	110	1407	42.001	16
A8806	2013	AUS (*5**,**10*)	0.5	2	16	4	>32	1	4	2	–	7363	1059	10	4015	64.001	227
H041	2009	Japan (*2*)	2	4	16	2	>32	0.5	4	4	–	7363	2594	10	4220	37.001	226

*AUS, Australia; AZM, azithromycin; β-lac, β-lactamase; CEF, ceftriaxone; CFM, cefixime; CIP, ciprofloxacin; DEN, Denmark; GEN, gentamicin; MLST, multilocus sequence type; NG-MAST, *Neisseria gonorrhoeae* multi-antigen sequence type; NG-STAR, *Neisseria gonorrhoeae* sequence type for antimicrobial resistance; NA, not available; PCN, penicillin; PPNG, penicillinase-producing *N. gonorrhoeae*; ref, reference; SPX, spectinomycin; TET, tetracycline; +, positive; –, negative.

## Methods

We confirmed *N. gonorrhoeae* isolates by using matrix-assisted laser desorption/ionization time-of-flight mass spectrometry (Bruker Daltonics, Melbourne, Victoria, Australia; bioMérieux, Brisbane, Queensland, Australia). We determined antimicrobial susceptibilities of *N. gonorrhoeae* to ceftriaxone, penicillin, tetracycline, azithromycin, gentamicin, and ciprofloxacin by using Etest (bioMérieux) and spectinomycin by using the agar dilution method (*11*). We interpreted MIC on the basis of interpretive criteria from the Clinical and Laboratory Standards Institute (*12*): penicillin resistance (MIC ≥2.0 mg/L); tetracycline resistance (MIC ≥2.0 mg/L); ciprofloxacin resistance (MIC ≥1.0 mg/L); and spectinomycin resistance (MIC ≥128.0 mg/L). Because the Clinical and Laboratory Standards Institute does not have an azithromycin breakpoint, and ceftriaxone breakpoints only state susceptibility (≤0.25 mg/L), we used the European Committee on Antimicrobial Susceptibility Testing (*13*) breakpoints for ceftriaxone resistance (MIC>0.12 mg/L) and azithromycin resistance (MIC>0.5 mg/L). β-lactamase production was analyzed by using nitrocefin (Thermo-Fisher Scientific, Melbourne, Victoria, Australia). We subcultured isolates on GC agar base with Vitox Supplement (Thermo-Fisher Scientific) and incubated for 24 h at 35°C in a 5% CO_2_ atmosphere with or without antimicrobial drugs and stored in Tryptone (Thermo-Fisher Scientific) soya broth with 10% glyercol at −80°C.

### Genomic Analyses

We put each isolate from Japan and Australia through DNA extraction, library preparation, and sequencing (Illumina, San Diego, CA, USA). From the strains from Japan, FC428 and FC460, we extracted DNA samples with the DNeasy Blood & Tissue Kit (QIAGEN, Tokyo, Japan). We created multiplexed libraries with Nextera XT DNA sample prep kit (Illumina) and generated paired-end 300-bp indexed reads on the Illumina MiSeq platform (Illumina) yielding 6,121,575 reads/genome and genome coverage of 845× for FC428 and 1,272,909 reads/genome and genome coverage of 845× for FC460.

To analyze the strains from Australia, A7536 and A7846, we extracted DNA on the QIAsymphony SP (QIAGEN) by using the DSP DNA Mini Kit (QIAGEN). We prepared the libraries according to manufacturer instructions for the Nextera XT library preparation kit (Illumina) and sequenced on the NextSeq 500 (Illumina) by using the NextSeq 500 Mid Output V2 kit (Illumina). Sequencing generated 6,763,774 reads and genome coverage of 361× for A7536 and 3,672,072 reads and genome coverage of 202× for A7846.

We then provided sequencing data to the Canadian National Microbiology Laboratory, where bioinformatic analyses were performed as previously described (*14*). Quality reads were assembled by using SPAdes (*15*) (http://bioinf.spbau.ru/spades) and annotated with Prokka (*16*) (https://github.com/tseemann/prokka), and produced an average of 86 contigs per isolate, an average contig length of 26,276 nt, and an average N50 length of 68,884 nt. Quality metrics for whole-genome sequencing (WGS) are shown in Technical Appendix Table 1. A core single-nucleotide variation (SNV) phylogeny was created by mapping reads to FA1090 (GenBank accession no. NC_002946.2) by using a custom Galaxy SNVPhyl workflow (*17*). Repetitive and highly recombinant regions with >2 SNVs per 500 nt were removed from the analysis. The percentage of valid and included positions in the core genome was 97.6%; 567 sites were used to generate the phylogeny. We used a meta-alignment of informative core SNV positions to create a maximum-likelihood phylogenetic tree for A7536, A7846, FC428, FC460, and 47707 (Figure). The H041, F89, and A8806 ceftriaxone-resistant strains (available in the World Health Organization [WHO] reference panel as WHO-X, WHO-Y, and WHO-Z, respectively) (*10*) were included for comparison. WGS read data for A7536, A7846, FC428, and FC460 are available under BioProject PRJNA416507, and previously reported 47707 was submitted under BioProject PRJNA415047 (*8*) .

**Figure:**
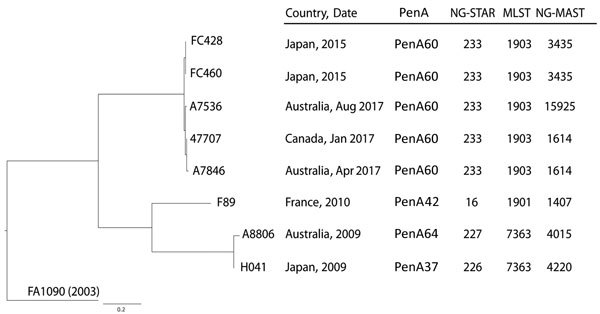
Core single-nucleotide variation (SNV) phylogenetic tree of ceftriaxone-resistant *Neisseria gonorrhoeae* isolates*.* The maximum-likelihood phylogenetic tree is rooted on the reference genome of *N. gonorrhoeae* FA1090 (GenBank accession no. NC_002946.2). Isolates are indicated by country and year. Strains F89, A8806, and H041 (World Health Organization [WHO] reference panel WHO-Y, WHO-Z, and WHO-X, respectively) are previously reported ceftriaxone-resistant reference strains (*10*). Scale bar indicates estimated evolutionary divergence between isolates on the basis of average genetic distance between strains (estimated number of substitutions in the sample/total number of high-quality SNVs). MLST, multilocus sequence type; NG-MAST, *Neisseria gonorrhoeae* multiantigen sequence type; NG-STAR, *Neisseria gonorrhoeae* sequence type for antimicrobial resistance; PenA, penicillin-binding protein 2.

We implemented *N. gonorrhoeae* multiantigen sequence typing (NG-MAST) (*18*), multilocus sequence typing (MLST) (*19*), and *N. gonorrhoeae* sequence typing for antimicrobial resistance (NG-STAR) (*20*) by using gene sequences extracted in silico from WGS data. We submitted the sequences to the NG-MAST (http://www.ng-mast.net/), *Neisseria* MLST (http://pubmlst.org/neisseria/), and NG-STAR (https://ngstar.canada.ca) databases to determine respective sequence types. Sequence data for the GK124 strain (*9*) were not available for these analyses; however, a summary of the documented susceptibility and MLST and NG-MAST data is provided (Table 1).

## Results

### Case Histories and Isolate Details

The first documented case-patient in Australia was a man in his forties who was visiting from the Philippines. He went to a sexual health clinic in Adelaide in April 2017 reporting urethral discharge and dysuria. He reported recent heterosexual contact with multiple female sex workers in Cambodia and the Philippines; it was unclear where the infection was acquired. An *N. gonorrhoeae* isolate (A7846) of similar susceptibility to FC428 (showing the characteristic ceftriaxone resistance and PPNG; Table 1) was cultured. The patient was treated with a 1-time dose combination therapy of ceftriaxone (500 mg IM) and azithromycin (1 g orally). A test result 7 days after treatment was negative for *N. gonorrhoeae*.

A second case-patient in Australia was a man visiting from China. He was in his early 40s and described symptoms of urethral discharge and dysuria to a general practitioner in Sydney in August 2017. He reported heterosexual contact in China, but none in Australia. An isolate (A7536) of similar susceptibility to FC428 (ceftriaxone-resistant and PPNG; Table 1) was cultured. The patient was treated with a 1-time dose combination therapy of ceftriaxone (500 mg IM) and azithromycin (1 g orally); he returned to China shortly thereafter. Attending physicians advised him to return to follow up for test of cure and to trace contacts, but follow-up was not confirmed.

Core SNV phylogenetic analysis results (Figure) showed a close genetic relatedness among the FC428, FC460, 47707, A7536, and A7846 isolates. These isolates were distinct from the other previously described F89, A8806, and H041 ceftriaxone-resistant strains; the 2 groups of isolates were separated from each other by an average of 292 core SNVs. We detected no SNVs in the 2 isolates from Japan (FC428, FC460 collected from the same patient 3 months apart). For other isolates, 12 SNVs separated FC428 from both 47707 and A7536; 17 SNVs separated FC428 and A7846 (47707, A7536, and A7846 shared 8 identical SNVs); 8 SNVs separated 47707 and A7536; 5 SNVs separated 47707 and A7846; and 11 SNVs separated A7536 and A7846 (Technical Appendix Table 2).

Molecular typing of FC428, FC460, 47707, A7536, and A7846 from the WGS showed an identical MLST of ST1903, which was also reported for GK124 from Denmark (*9*) (Table 1). We observed different NG-MAST: ST3435 for FC428 and FC460; ST1614 for 47707, A7846, and GK124; and ST15925 for A7536. FC428, FC460, 47707, A7536, and A7846 were of the same NG-STAR, ST233, which was characterized by a mosaic *penA*-60.001 allele that had only been reported previously for FC428 and 47707 (*8*). This allele encodes key alterations A311V and T483S in PBP2 (Table 2; also observed for GK124) that are linked to ceftriaxone resistance, some of which are also present among the previously described ceftriaxone-resistant strains (Table 2 [*1*]). We observed additional resistance mutations for FC428, FC460, 47707, A7536, and A7846 by using the NG-STAR designations, including the previously described alleles *mtrR*-1 (promoter−35A deletion); *porB*-8 (PorB G120K, PorB A121D); *ponA*-1 (PonA L421P); *gyrA*-7 (GyrA S91F, GyrA D95A); and *parC*-3 (ParC S87R).

**Table 2:** PenA types identified in ceftriaxone-resistant *Neisseria* *gonorrhoeae* strains*

PenA type	Strain ID	Amino acid position in PenA protein (*2**,**18*)
		34711111222222222223333333333333333333333333344444444444444444444555555555555555555 51000467000013678891112222223333444445777788800011134456666677888001111344455555677 01403123440295811263467890125123563467836914702384882356903146251367323602367756 ↑ ↑ ↑
0	M32091	MCAKDDVNYGEDQQAADRRAIVAGTDLNERLQPSPR.SRGAEFEITLNRRPAVLQIFESRENPTTAFANVAAHGGAPPKII.A
37	H041	....E.ASHAGEE..VEKQVMPS.V.TTDTFL.ATQ.TMTPK.DVSV.K..VEVKVIA.KKEASI.LVY...N.ST.VQVVNV
42	F89	....E.ASHAGEE..VEKQ.MTS.V.ATDTFLSATQ.TMTPK.DV..S.QKVEVKVIA.KKEA..PLVY...N.S........
60	FC428/ FC460/ A7536/ A7846/ 47707	...................VMTS.V.PTDTFL.ATQ.TMTPK.DV..S.QKVEVKVIA.KKEASI.LVY...N.ST.VQVVNV
64	A8806	....E.ASHAGEE......VMTS.V.PTDTFL.ATQ.TMTPK.DV..S.QKVEVKVIA.KKEASI.LVY...N.ST.VQVVNV

*Arrows indicate key amino acid positions associated with high level β-lactam resistance. PenA, penicillin-binding protein 2.

## Discussion

The recent reports of the *N. gonorrhoeae* FC428 clonal strain in Denmark, Canada, and now Australia provide new evidence that there is sustained international transmission of a ceftriaxone-resistant *N. gonorrhoeae* strain. This strain appears to have been circulating globally for >2 years. Thus, it is highly likely this strain is prevalent elsewhere, possibly in Asia, but undetected. There are serious gaps in *N. gonorrhoeae* antimicrobial resistance surveillance worldwide (*21*), and we estimate that samples from as few as 0.1% of the estimated 80 million cases of *N*. *gonorrhoeae* reported globally each year (*22*) are tested for antimicrobial resistance. Therefore, there are many opportunities for such strains to avoid detection.

Fortunately, the ceftriaxone MICs of the FC428 clonal strain remain lower than the H041 strain from Japan (MIC 2 mg/L) (*2*), and further, the FC428 strain does not exhibit resistance to azithromycin (Table 1). Therefore, treatment failure is arguably less likely against FC428 infections than in H041 and F89 infections, particularly when using ceftriaxone and azithromycin dual therapy; treatment failure was not observed in our study. Nevertheless, previous pharmacodynamic analyses indicate that ceftriaxone MICs of 0.5–1.0 mg/L can result in treatment failures with ceftriaxone 250 mg monotherapy and even (albeit to a lesser extent) when 1.0 g doses are used (*23*). As such, a dissemination of the FC428 clone could offset dual therapy guidelines because azithromycin resistance is being increasingly reported (*24**,**25*).

The cases of *N*. *gonorrhoeae* described here and the circumstances under which these analyses took place are also a timely reminder of the need for international collaboration in addressing the overall *N*. *gonorrhoeae* problem and highlight the benefits of rapid access to genomic data by using electronic communications. In fact, in the absence of WGS data, it would have been very difficult to identify the links between these isolates. Not only have we been able to use these tools to readily identify the problem but we also arguably achieved identification in a sufficiently timely manner as to enable countries to put in place interventions that can limit further the spread of this strain, including intensifying follow-up and contact tracing. 

Differences in extraction and sequencing procedures among the 3 countries could introduce variations in DNA concentrations that might affect the quality of the sequencing, such as number of reads and depth of coverage. This limitation was minimized because downstream processing of the data, such as assembly and reference mapping software algorithms, standardizes input data before detailed analyses of the genomes are conducted. Laboratory and epidemiologic findings are critical for surveillance that closely tracks the dissemination and emergence of epidemic antimicrobial-resistant strains and for rapid recognition and implementation of control measures to limit the expansion of clones through sexual networks. We recommend that health departments in all countries be made aware of this spreading resistant strain and strengthen *N*. *gonorrhoeae* antimicrobial-resistance monitoring, including treatment failure identification, adequate follow-up and contact tracing of cases, and STI prevention programs.

In conclusion, international collaboration based on WGS typing methods revealed the dissemination of a ceftriaxone-resistant *N. gonorrhoeae* in Japan, Canada, and Australia. Sustained transmission spanning 2 years suggests unidentified cases are likely present in other locations. These findings warrant the intensification of surveillance strategies and establishment of collaborations with other countries to monitor spread and inform national and global policies and actions.

Technical Appendix Whole-genome assembly and fast quality control sequencing metrics of and core single-nucleotide variations between *Neisseria* *gonorrhoeae* isolates.
