# Supplementary material for: Cooperative Recognition of Internationally Disseminated Ceftriaxone-Resistant Neisseria gonorrhoeae Strain
Source: Emerg Infect Dis. 2018 Apr;24(4):735–40. doi: 10.3201/eid2404.171873 (PMC5875269; doi:10.3201/eid2404.171873)
Supplement: Technical Appendix — Whole-genome assembly and fast quality control sequencing metrics of and core single-nucleotide variations between Neisseria gonorrhoeae isolates. [file 17-1873-Techapp-s1.pdf]

Article DOI: https://doi.org/10.3201/eid2404.171873

Cooperative Recognition of Internationally Disseminated Ceftriaxone-resistant *Neisseria gonorrhoeae* Strain

Technical Appendix

Technical Appendix Table 1. Whole genome assembly and fast quality control sequencing metrics.

Assembly metrics

Strain	Min contig length	Max contig length	Mean contig length	Standard deviation of contig length	Median contig length	N50 contig length	No. contigs	No. contigs >=1kb	No. contigs in N50	No. bases in all contigs	No. bases in contigs >=1kb	GC Content of contigs
A7536	503	153179	20434.6	27096.1	9761	46889	107	95	15	2186502	2178363	52.34%
A7846	508	170175	19717.04	27713.47	8163	47017	111	93	15	2188591	2175598	52.35%
FC428	530	205307	28134.55	36437.23	13956	60704	78	67	12	2194495	2186878	52.31%
FC460	530	221468	20948.54	31872.34	8474	47483	104	90	14	2178648	2169052	52.49%
H041	510	337256	29213.03	49753.63	7503	86362	72	57	8	2103338	2092530	52.64%
F89	500	260748	29941.97	45758.24	9726	73376	72	59	9	2155822	2146570	52.40%
A8806	510	222588	33184.75	51000.25	7449	112376	65	57	7	2157009	2150822	52.40%
47707	647	208505	28633.58	45486.76	7609	76870	77	64	8	2204786	2193928	52.34%

FastQC Metrics

SE/PE	Encoding	No. Reads	Total no. Base Pairs	Sequence length range	Most abundant read length	No. reads for abundant	Estimated Coverage	Reference length	Duplicate % R1	Duplicate % R2	No. overrepresented sequences
PE	Sanger / Illumina 1.9	6763774	777480038	35-151	150	2793366	360.96	2153922	63.98	62.11	0
PE	Sanger / Illumina 1.9	3673072	435717067	35-151	150	1648675	202.29	2153922	57.22	55.61	0
PE	Sanger / Illumina 1.9	6139112	1820565211	35-301	300	5438042	845.23	2153922	75.42	75.19	0
PE	Sanger / Illumina 1.9	1278242	351830155	35-301	300	977031	163.34	2153922	48.85	48.54	0.22
PE	Sanger / Illumina 1.9	521860	156934157	85-301	300	497560	72.86	2153922	9.9	7.17	0
PE	Sanger / Illumina 1.9	529020	159081437	35-301	300	503799	73.86	2153922	17.81	15.52	0
PE	Sanger / Illumina 1.9	851030	255904621	46-301	300	811357	118.81	2153922	11.23	8.54	0
PE	Sanger / Illumina 1.9	697922	209832768	48-301	300	664719	97.42	2153922	11.57	10.36	0

Technical Appendix Table 2. Number of core SNVs among *Neisseria gonorrhoeae* isolates*

Isolate	FC428	FC460	A7536	47707	A7846	F89	A8806	H041	FA1090
FC428	0	0	12†	12†	17†	275	305	292	307
FC460	0	0	12†	12†	17†	275	305	292	307
A7536	12†	12†	0	8	11	281	305	292	311
47707	12†	12†	8	0	5	281	305	292	309
A7846	17†	17†	11	5	0	280	306	293	308
F89	275	275	281	281	280	0	236	225	322
A8806	305	305	305	305	306	236	0	17	352
H041	292	292	292	292	293	225	17	0	339
FA1090	307	307	311	309	308	322	352	339	0

*SNV, single nucleotide variation.
†8 identical SNVs. Sample numbers listed in order as they appear in the phylogenetic tree of Figure 1.
